# Intrapersonal synchrony analysis reveals a weaker temporal coherence between gaze and gestures in adults with autism spectrum disorder

**DOI:** 10.1038/s41598-022-24605-8

**Published:** 2022-11-27

**Authors:** Carola Bloch, Ralf Tepest, Mathis Jording, Kai Vogeley, Christine M. Falter-Wagner

**Affiliations:** 1 Department of Psychiatry and Psychotherapy, Medical Faculty, LMU Clinic, Ludwig-Maximilians-University, Nussbaumstraße 7, 80336 Munich, Germany; 2 Department of Psychiatry and Psychotherapy, Faculty of Medicine and University Hospital Cologne, University of Cologne, Cologne, Germany; 3 Cognitive Neuroscience, Institute of Neuroscience and Medicine (INM-3), Forschungszentrum Jülich, Jülich, Germany

**Keywords:** Human behaviour, Psychiatric disorders, Autism spectrum disorders, Psychology, Signs and symptoms, Social behaviour

## Abstract

The temporal encoding of nonverbal signals within individuals, referred to as intrapersonal synchrony (IaPS), is an implicit process and essential feature of human communication. Based on existing evidence, IaPS is thought to be a marker of nonverbal behavior characteristics in autism spectrum disorders (ASD), but there is a lack of empirical evidence. The aim of this study was to quantify IaPS in adults during an experimentally controlled real-life interaction task. A sample of adults with a confirmed ASD diagnosis and a matched sample of typically-developed adults were tested (*N* = 48). Participants were required to indicate the appearance of a target invisible to their interaction partner nonverbally through gaze and pointing gestures. Special eye-tracking software allowed automated extraction of temporal delays between nonverbal signals and their intrapersonal variability with millisecond temporal resolution as indices for IaPS. Likelihood ratio tests of multilevel models showed enlarged delays between nonverbal signals in ASD. Larger delays were associated with greater intrapersonal variability in delays. The results provide a quantitative constraint on nonverbal temporality in typically-developed adults and suggest weaker temporal coherence between nonverbal signals in adults with ASD. The results provide a potential diagnostic marker and inspire predictive coding theories about the role of IaPS in interpersonal synchronization processes.

## Introduction

Autism spectrum disorder (ASD) entails difficulties in social interactions and communicative behavior as main diagnostic features. Empirically, this is supported by reduced interpersonal synchrony^1–4^. However, behavioral measures that can serve as indicators for (mis-) alignment between individuals are not yet defined.

There are heterogeneous findings concerning the altered amounts of communicative behavior in ASD. Studies showed either reduced^5^, similar^1,3,6,7^, or increased amounts of communicative behavior^8^. As such, a diagnostic emphasis has been put on qualitative aspects or the manner of communicative behavior in ASD. Aside from differentiated motor abilities, which are frequently reported in ASD^9–13^, alterations in the timing of communication signals could be constitutive for observations of interpersonal divergence^14–16^. Nonverbal timing mechanisms in interactions are acquired from an early age through interactions between caregivers and children^17–20^ and manifest in adulthood as a mostly implicit and automatic process (i.e., we do not actively think about how to time a gesture with gaze). This implicit temporal configuration of nonverbal signals within an individual is from here on termed intrapersonal synchrony (IaPS).

Despite equal amounts of communication behavior, one study showed increased asynchrony between gestures and co-occurring speech within individuals, as indicated by larger intervals between semantic aspects of speech and gesture events, in adolescents with ASD^6^. Furthermore, the communicative quality of narratives produced by individuals with ASD (as judged by non-autistic raters) was reduced, and while the number of gestures was positively correlated with communicative quality in the comparison group, this was not the case for the ASD group. Besides this empirical evidence, alterations of IaPS in ASD are anecdotally well-known in the clinical setting and are part of the ADOS-G coding system^21^; for example, in the use of emphatic gestures (i.e., gestures that are temporally aligned with speech emphasis) in item A10 or the coordination of gestures (e.g., with gaze) in item B3.

Although alterations in IaPS in ASD are to be expected and are diagnostically relevant, an experimental investigation and quantification of the differences is still pending. Thus far, evidence is limited to intrapersonal asynchronies between semantic aspects of speech and gestures^6^, so if and how deviations are also present in the nonverbal domain remain open questions. An examination of IaPS in the nonverbal domain is particularly intriguing given the important role of gaze during social interactions^22–24^, the extensive evidence for alterations of social gaze behavior in ASD^25–28^, and considerations that the temporal integration of gaze with other nonverbal behavior is an important mechanism during social interactions^29,30^.

### IaPS in deixis

Considering the concept of deixis in the nonverbal domain, gaze and pointing signals are temporally integrated to establish a spatial reference from a person´s perspective in relation to another person^31,32^. Deictic signals are fundamental building blocks of communication and serve the coordination of attention during interactions from infancy onward^33–35^. Directing a partner’s attention through deictic signals allows for the development of shared knowledge and “common ground”. During joint attention processes, one study showed that deictic gaze signals are automatically integrated with pointing signals and thereby support response behavior^36^. The ubiquitous use of deictic gaze and gesture signals and their automatic integration during joint attention processes underpins the suitability to investigate IaPS of deictic signals in this study.

### Purpose of the current study

In the current study, we segmented deixis into gaze and gestures in order to measure IaPS between both nonverbal signals. While participants were engaged in a real-life interaction task with another person, we measured communication signal onsets and analyzed their temporal alignment as an index of IaPS. In line with the finding of larger asynchronies of semantic aspects of speech and co-speech gestures in adolescents with ASD^6^ and evidence for larger temporal binding windows in ASD^37^, we tested the hypothesis that individuals with ASD produce increased gaze-gesture delays. In addition, we raised the hypothesis of enlarged within-subject variability of gaze-gesture delays in ASD, given empirical evidence for reduced sensitivity in temporal interval discrimination in ASD^38^.

## Method

### Participants

The project goal, study site, funding sources, and inclusion/exclusion criteria were preregistered prior to realization at the WHO-approved German register of clinical trials (accessible on www.drks.de via reference number: DRKS00011271). The project was approved by the ethics committee of the Medical Faculty of the University of Cologne (approval number: 16-126). All methods have been performed in accordance with regulations in the Declaration of Helsinki. All participants signed a written informed consent and were financially compensated for their participation. A power analysis prior to study realization was conducted in the program G*Power^39^ with the aim of a statistical effect size of 0.80, given an alpha level of 0.05, and an estimated effect size of *d* = 0.76, derived from our own previous synchrony study^38^. A sample size of 23 subjects per group was determined as sufficient to detect an existing effect under these criteria.

A sample of 28 individuals (11 identifying as female, 16 as male) diagnosed with F84.5 according to ICD-10^40^ was recruited at the outpatient clinic for autism in adulthood (Department of Psychiatry, University Hospital Cologne) from 2019 until 2021. One participant (18 years old) was recruited via the pediatric outpatient clinic for autism (University Hospital Cologne). Three participants from the ASD sample were excluded from the analysis due to ocular conditions that interfered with the eye-tracking system (i.e. nystagmus, cataract, and high diopter [> 18]), and one participant with ASD opted to discontinue the experiment early.

The final ASD group consisted of 24 individuals (10 identifying as female, 14 as male), aged *M* = 40.25 (range: 18–59). All diagnostic decisions were made in accordance with German S3 guidelines for diagnostics of ASD^41^. A gender-, age-, handedness-, and IQ-matched control sample consisting of 24 typically-developed (TD) participants, aged *M* = 36.67 (range: 19–58) was recruited online via social media platforms and advertisement in the University Hospital Cologne intranet.

Inclusion criteria were age between 18 and 60, normal or corrected to normal vision, no psychiatric or neurologic disorders, no current psychoactive medication, and written informed consent. For individuals from the diagnosis group, in addition to autism, depression and antidepressant use were not exclusion criteria due to their high prevalence in ASD populations, and our aim to include a representative sample^42,43^. Exclusion criteria were individuals not meeting the age criterion, current or prior diagnosis of neurological disorders, acute suicidality, danger to self or others, and lack of or inability to provide written informed consent. In addition to the preregistered exclusion criteria, motor impairment in arm movements was added as an exclusion criterion for both groups to ensure unimpeded execution of the gestures. Eleven participants reported current or past depressive episodes, and seven of these participants reported medication with antidepressants (see Supplementary Material, Supplementary Fig. 1 for further examination).

### Sample pretests

Participants completed a battery of tests prior to the IaPS experiment, consisting of a demographic data questionnaire and a sensorimotor synchronization tapping task (this is mentioned for completeness, but the data from this task are not further discussed here).

In addition, German versions of clinical screening instruments were conducted to compare the groups with respect to autism-typical characteristics: The *Autism-Spectrum Quotient* (AQ)^44^ assessed autistic traits, the *Empathy Quotient* (EQ)^45^ assessed empathy levels, the *Systemizing Quotient* (SQ)^46^ assessed systemizing styles, and a 24-Item version of the *Reading the Mind in the Eyes* test (RME)^47,48^ assessed mentalizing with emotional demand^49^. The *Sensory Perception Quotient* (SPQ)^50^ was used to assess sensory sensitivity, and the *Adult Developmental Co-ordination Disorders/Dyspraxia Checklist* (ADC)^51^ assessed motor coordination difficulties. *Beck’s Depression Inventory* (BDI)^52^ was carried out in order to evaluate depressive symptomatology.

Further neuropsychological testing was performed to compare groups on these dimensions: The *D2*^53^ classified concentration abilities. The *Wortschatztest* (WST)^54^ and the *Wechsler Adult Intelligence Scale* (WIE-III)^55^ provided IQ scores.

Sample characteristics and group comparisons are shown in Table 1. Regarding the clinical screening tools, the groups differed significantly on AQ, EQ, and SQ. In addition, the ASD group presented with significantly higher depressive symptoms (BDI), as well as higher dyspraxia scores (ADC). The groups did not differ on sensory sensitivity (SPQ) and mentalizing with emotional demand^49^ (RME).

Regarding ﻿the neuropsychological tests, the groups scored similarly on verbal IQ (WST; VIQ), performance-related IQ (WIE-III; PIQ), and concentration ability (D2).

**Table 1:** Sample characteristics and group comparisons.

Measure	M_ASD_	SD_ASD_	M_TD_	SD_TD_	Statistic	*p*	Eff. size
Age	40.84	12.12	37.05	12.66	*t*(46) = 1.06	0.296	*d* = 0.31
AQ	41.50	3.81	14.61	5.87	*t*(37.5) =18.55^ab^	< .001	*d* = 5.44
EQ	11.96	5.38	47.96	10.79	*t*(32.0) =− 14.38^ab^	< .001	*d *= − 4.22
SQ	44.83	13.75	20.61	10.34	*U* = 500^c^	< .001	*r* = 0.70 ^c^
RME	16.38	4.45	18.62	3.02	*U* = 218^c^	.146	*r* = 0.21 ^c^
SPQ	59.92	17.48	60.04	17.22	*t*(45) = − 0.03^b^	.980	*d* = − 0.01
ADC	51.00	12.83	22.35	12.41	*U* = 516^cb^	< .001	*r* = 0.75 ^c^
BDI	15.88	10.73	5.50	5.48	*U* = 448^c^	< .001	*r* = 0.48 ^c^
WST	111.46	11.74	110.67	8.35	*t*(46) = 0.27	.789	*d* = 0.08
D2	103.83	9.50	100.04	7.79	*t*(46) = 1.51	.137	*d* = 0.44
IQ	114.38	16.48	108.50	13.07	*t*(46) = 1.37	.178	*d* = 0.40
PIQ	110.67	17.65	102.38	13.66	*t*(46) = 1.82	.080	*d* = 0.53
VIQ	115.00	15.95	112.17	12.61	*t*(46) = 0.68	.498	*d* = 0.20
YoE	20.12	5.59	18.46	4.84	*U *= 349^c^	.211	*r* = 0.18

Means (*M*) with standard deviations (*SD*) in the ASD group and the TD group with results of two-sided Student’s *t* tests (*α* = 0.05) and Cohen’s *d* as effect sizes.

Autism quotient (AQ), Empathy quotient (EQ), Systemizing quotient (SQ), Reading the mind in the eyes test (RME), Sensory perception quotient (SPQ), Adult dyspraxia checklist (ADC), Beck’s depression inventory (BDI), Wortschatztest, (WST) Concentration ability test (D2), Intelligence quotient (IQ), Performance-based intelligence quotient (PIQ), Verbal intelligence quotient (VIQ), Years of education (YoE).

^a^Bartlett test revealed heterogeneous variances and results of Welch’s two-sample *t* test are reported.

^b^Data from one control participant missing.

^c^Shapiro-Wilk test revealed non-normality of data in groups and results of Mann–Whitney test with effect size (*r*) are reported.

### Task setup

**Figure 1:**
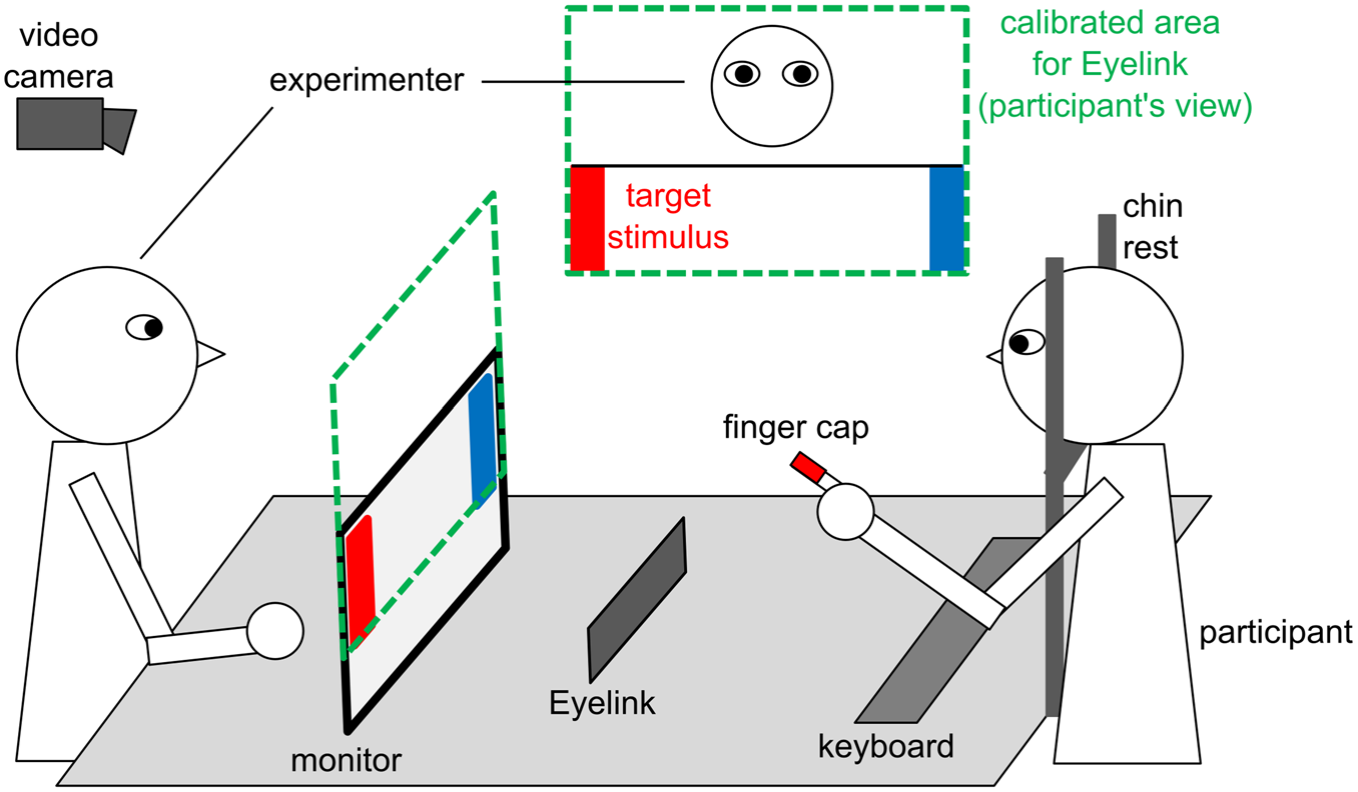
Schematic display of the experimental task setup during free task version and nonverbal task version. The experimenter was the designated interaction partner during the tasks and sat opposite of the participants. A monitor facing the participants displayed the stimuli. An Eyelink recorded participants’ eye movements, and the calibrated area included the interaction partner’s facial area and the stimuli. In the free task version, participants were free to choose how to convey the spatial information of the target stimulus to the partner. In the nonverbal task version, participants were instructed to press the space bar of a keyboard located in front of them and release it only for the pointing gesture. A video camera recorded all test runs, and the colored finger caps that participants wore on their index fingers were used to later extract the movement trajectories (see “Video analysis”).

The experiment was conducted in a quiet, windowless room with stable light conditions. Participants were seated at a table opposite to the experimenter (see schematic display in Fig. 1). A chin and forehead rest inhibited major head movements during eye-tracking. A HP E241i LED monitor with a refresh rate of 60 Hz and 1920 × 1200 resolution was placed 94 cm in front of the participants. The monitor position was lowered maximally during the trials in order to assure no visual barrier between the experimenter and participants. A HP keyboard was placed in front of the participants. A Microsoft HD lifecam camera and a Logitech C270 camera were placed on the wall behind the experimenter. Due to technical reasons, one camera was used in the first task version and the other in the second task version (task versions are described below). Both cameras recorded the participants in 30 fps from an approximate 60-degree angle. A Zoom H4 microphone was used for voice recordings. Participants were given colored finger caps applied in a suitable size on their index fingers for later extraction of movement trajectories (see “Video analysis”).

### Design and procedure

All participants were instructed to engage in an interaction with the experimenter (CB). In each trial, a blue and a red bar appeared on the sides of the upper half screen in a randomized and counterbalanced order for all blocks. The participant’s task was to let their interaction partner know where the red target appeared. Participants were told that the task of the interaction partner was to note their responses. Each trial started with a tone after which participants – as soon as they felt ready – had to establish eye-contact with their interaction partner to signalize mutual readiness. After eye-contact was established, the experimenter initiated the stimulus presentation via mouse-click.

Two task versions were conducted in the same order for all participants: First, participants were instructed that they are free to choose how they inform their interaction partner, that their response does not depend on reaction time, and that they should behave most natural and intuitive. This task version assessed the spontaneous use of communication channels and will be referred to as the *free task version* in the following. Second, participants were instructed to indicate the position of the red bar with gaze and pointing gestures. In between trials, participants pressed down the space bar with the index finger of their dominant hand and were instructed to only release it to perform the pointing gesture. An instruction video was shown to ensure that the task was understood correctly. This task was used to assess the temporal alignment of nonverbal signals as an index of IaPS and will be referred to as the *nonverbal task version* in the following. Each task version consisted of 10 practice trials and four blocks á 30 trials with short breaks between the blocks. The experimental script that was used for data acquisition is publicly available at the Open Science Framework (https://osf.io/t4wmr/). Participants took part in another unrelated study on the same day, but the order in which the experiments were conducted was randomized with a break in between.

### Modifications due to pandemic regulations

Due to hygiene requirements posed by the Covid-19 pandemic in 2020, we had to incorporate a glass pane between the interaction partners. To minimize reflections, an anti-reflection foil has been applied to the pane, and the screen background was changed from grey to white. Fourteen participants with ASD and 17 TD participants were tested with this modified setup (see Supplementary Material, Supplementary Fig. 2 for examinations). The modified script version is publicly available at https://osf.io/t4wmr/.

### Additional measures

We included two additional measures besides the indices of IaPS: First, regarding potential differences in the quantities of communication behavior^5,8^, we investigated the spontaneous use of communication channels including verbal output during the free task version. Second, to study motor atypicalities in ASD that may potentially influence IaPS^9–13^, we captured movement trajectories of the gestures and examined potential group differences in spatio-temporal features during the nonverbal task version. These could be included as covariates to control for possible spatial differences in the analysis of temporal parameters.

### Eye-tracking and data synchronization

An Eyelink 1000 Plus system (SR Research Ltd., Hamilton, Ontario, Canada) recorded monocular eye movements in a pupil-CR tracking mode with a 1000 Hz temporal resolution. The tracker was controlled via a connection with the software PsychoPy^56^. Events from external devices were time-locked in the time-series gaze data using features of the PyLink module. The Eyelink systems online parser was used to detect gaze events within the sample data. The velocity threshold for saccade detection of the parser was 30°/second. A 9-point calibration and validation were carried out before each task version. This allowed for the definition of regions of interest (RoI) by means of the setup dimensions in the visual space of the participants: The social RoI was defined as a square that covered the face of the interaction partner, and the stimuli RoIs covered the red and blue bars on the screen.

### Gaze selection algorithm

To investigate gaze-gesture delays, communicative gaze shifts had to be identified for each trial. Communicative gaze shifts were selected from the gaze data by applying a selection algorithm in R^57^. Communicative gaze shifts were defined as starting in the social RoI and ending in the target RoI. Thereby, these gaze shifts served a communicative function, guiding the attention of the interaction partner as in joint attention processes^58,59^. Three different gaze pathways were possible: direct saccades, saccade pathways with an intermediate fixation of the social RoI (second saccades were selected), or saccade pathways with an intermediate fixation of a random RoI (first saccades were selected). This tolerance in the pathways considers that eye movements are spatially inaccurate to a certain degree (i.e. hypo- and hypermetric)^60–62^ and are potentially embedded in saccade chains (see Supplementary Material, Supplementary Table 1 for frequencies of pathways and Supplementary Fig. 3 for investigations). Further exclusion criteria for communicative gaze shifts selection were applied (see Supplementary Material, Supplementary Table 2 for number of exclusions per category in both groups):

1. First trials of each block were excluded due to recommendations of the eye-tracking system.
2. As the instruction was to establish eye-contact in the beginning of each trial, we excluded trials in which gaze was not in the social RoI at stimulus onset.
3. Trials without communicative gaze shifts were excluded.
4. Corresponding to the procedure in Johnson et al.^63^, trials were excluded in which participants blinked < 100 ms before stimulus onset.
5. Trials were excluded in which the saccade latency was too short to be externally triggered (< 75 ms, in accordance with Bibi & Edelman^64^).
6. Trials were excluded in which the gesture onset preceded saccade onsets because of low incidences and gaze was shown to be the preceding signal in studies of eye-hand-coordination^32,65–69^.

For each trial, the first communicative gaze shift after stimulus onset that met the aforementioned requirements was identified by the algorithm.

### Data preprocessing

For the free task version, video and voice recordings were annotated by two independent raters, naïve to diagnosis. One rater evaluated all sound recordings and documented the spontaneous usage of verbal utterances as a binary variable (voice = 1, no voice = 0). The other rater similarly documented spontaneous gesture usage by assessing the video recordings and creating a binary variable (gesture = 1, no gesture = 0). A third binary variable for gaze (gaze = 1, no gaze = 0) was created by applying the gaze selection algorithm and coding incidences of communicative gaze shifts as 1. Trials that were dismissed by the algorithm were coded as 0. Based on this, six binary variables were created that logged the appearance of spontaneous (multi-) channel usage per channel(s) per trial: gaze-only, verbal-only, gesture-only, gaze-and-gesture, gaze-and-verbal, verbal-and-gesture. In addition, three bivariate variables were created that logged the number of channels used, independent of the channels modality.

Due to a technical issue, all data from the free task version were lost for one participant with ASD. In the comparison group, data from three TD participants were partially lost (16, 12, and 7 trials respectively), and in the ASD group, data from one participant were partially lost (2 trials). For one participant from the TD group, the gaze data were erroneous for the free task version and incidences of spontaneous gaze were annotated manually from the video recordings. As first trials were excluded by the gaze selection algorithm, these were excluded from the final data as well, leaving 5415 trials (n = 2666 from the ASD group) for analysis.

For the nonverbal task version, trials were only included in the analysis in which a communicative gaze shift was selected. One trial from one TD participant was additionally excluded due to an error value on a control measure. For each of these trials, the onsets of communicative gaze shifts were subtracted from the onsets of the pointing gestures to derive the gaze-gesture delay as an index of IaPS. In total, 4563 trials (n = 2235 from the ASD group) were analyzed for the nonverbal task version. Missing data were handled by deploying adequate statistical methods (i.e., mixed effects models).

### Video analysis

The video recordings were used as input to automatically assess spatio-temporal features from the gestures. The video analysis was implemented by applying an in-house script generated with Matlab R2017b inclusive Image Processing Toolbox (The MathWorks Inc., Natick, MA). A red finger cap was applied to participants’ left index finger and a blue finger cap was applied to the right index finger. This allowed the trajectories to be extracted separately per side. Within a manually defined image RoI, every video was automatically analyzed frame by frame. The image of each frame was divided into RGB color components. Disturbances due to technical artifacts were reduced by image filter operations. Characteristic numerical values of intensity in the RGB components were used as threshold values for both finger cap colors to generate three intermediate binary images. These binary images served as masks for the segmentation of the fingertip targets. A function available with morphological operations was applied to shrink extended objects in images to points. Each point position was stored via its corresponding image plane coordinates. The procedure was performed frame by frame leading to two-dimensional time-series of data representing the gesture trajectories. This data was kernel-smoothed and vectorized, whereby each vector was aggregated into its amplitude and mean velocity. Nineteen trials from five participants without ASD and 10 trials from five participants with ASD were missing due to errors that inhibited the parsing of resting and movement in the data (e.g., masking of finger caps).

### Statistical procedures

We used the open source software R version 4.0.3^57^ with Rstudio version 1.4.1103^70^ and packages integrated in the *tidyverse* library^71^ for data processing and analyses. Data were analyzed in multilevel models as recommended for data with repeated measures or nested data designs^72,73^. We fitted generalized mixed effects models (GLMM) and linear mixed effects models (LMM) with the *lme4* package^73^, applying the maximum likelihood method for estimation of coefficients. Significance at 0.05 level were tested via model comparisons of models with and without the factor in question. Therefore, likelihood ratio tests were conducted, testing the increase of model fit by incremental inclusions of factors while taking into account the model complexity. The *parameters* package was used to retrieve model parameters with confidence intervals and p-values based on Satterthwaite approximation for all coefficients^74^. We examined whether our models fulfilled relevant assumptions – namely a lack of multicollinearity, normality of residuals, and homoscedasticity – deploying the check_model() function from the package *performance*^75^, which created plots for visual inspection of all assumptions. We calculated Pearson correlations, Shapiro–Wilk tests, Mann–Whitney tests, Student’s *t *tests, Wald tests, and effect sizes using the *rstatix* package^76^. Visualizations were created with the package *ggplot2* from the *tidyverse*^71^ and the package *ggdist*^77^.

Post-hoc equivalence tests were performed for major non-significant group effects to draw conclusions about the null hypothesis (see Supplementary Material, Supplementary Analysis 1).

P-values for comparisons in the analysis of spontaneous channels use and trajectory analysis were each corrected for multiple comparisons^80^.

### Mixed models

For gaze-gesture delays as dependent variable, data were used in long format, so each row represented measurements in one trial. A first LMM was fitted that included experimental block and target side as fixed factors, with random intercepts for subjects and random slopes for blocks and target side. The random effects structure was chosen in alignment with recommendations from the literature^72,78,79^ and should account for variation clustered in subjects, as well as for individual differences in the impact of block (e.g., fatigue or learning effects) and target side. This model was compared to a LMM including group as an additional fixed factor. For analysis of SD of delays, the long data was aggregated into the SD of delays per subject per block per target side. One SD for one participant with ASD could not be calculated due to only one observation. Including random slopes for target side and blocks, likewise to the models for gaze-gesture delays, resulted in a failure to converge due to an insufficient number of observations after aggregation. Thus, a first LMM was fitted with random intercepts for subjects and fixed factors for experimental blocks and target position. The effects structure was chosen in order to account for repeated measurements and systematic variation that is accounted for by blocks and target sides. This LMM was compared to a second LMM containing group as additional fixed factor. For the free task version, all binary variables were analyzed separately as dependent variables in binomial GLMM with random intercepts for subjects. These models were compared to GLMM that additionally included group as fixed factor. For the trajectory analysis, LMM structures were similar to those for gaze-gesture delays.

## Results

### Spontaneous channel use

Results of the model comparisons showed non-significant increases in model fits by including the group factor for all (multi-)channel use variables: verbal-only (*χ*^2^(1) = 1.01, *p* = .769), gesture-only (*χ*^2^(1) = 0.24, *p* = .769), gaze-only (*χ*^2^(1) = 0.48, *p* = .769), gaze-and-gesture (*χ*^2^(1) = 0.04, *p* = .840), gaze-and-verbal (*χ*^2^(1) = 0.73, *p* = .769), gesture-and-verbal (*χ*^2^(1) = 0.56, *p* = .769). Likewise, this result was non-significant for the count of channels that were combined, independent of specific channels: unimodal (*χ*^2^(1) = 3.76, *p* = .471), bimodal (*χ*^2^(1) = 0.31, *p* = .769), and trimodal (*χ*^2^(1) = 0.17, *p* = .769).

For the GLMM with gaze-only, we encountered a case of complete separation (i.e., the outcome variable separated the predictor variable perfectly). This was due to the fact that there were no occurrences of gaze-only in the ASD group, which led to inflated standard errors for the group coefficient. Therefore, we recommend to treat this result with caution and base our inference on the model for unimodal channel use in which gaze-only is included.

### IaPS measures

**Figure 2:**
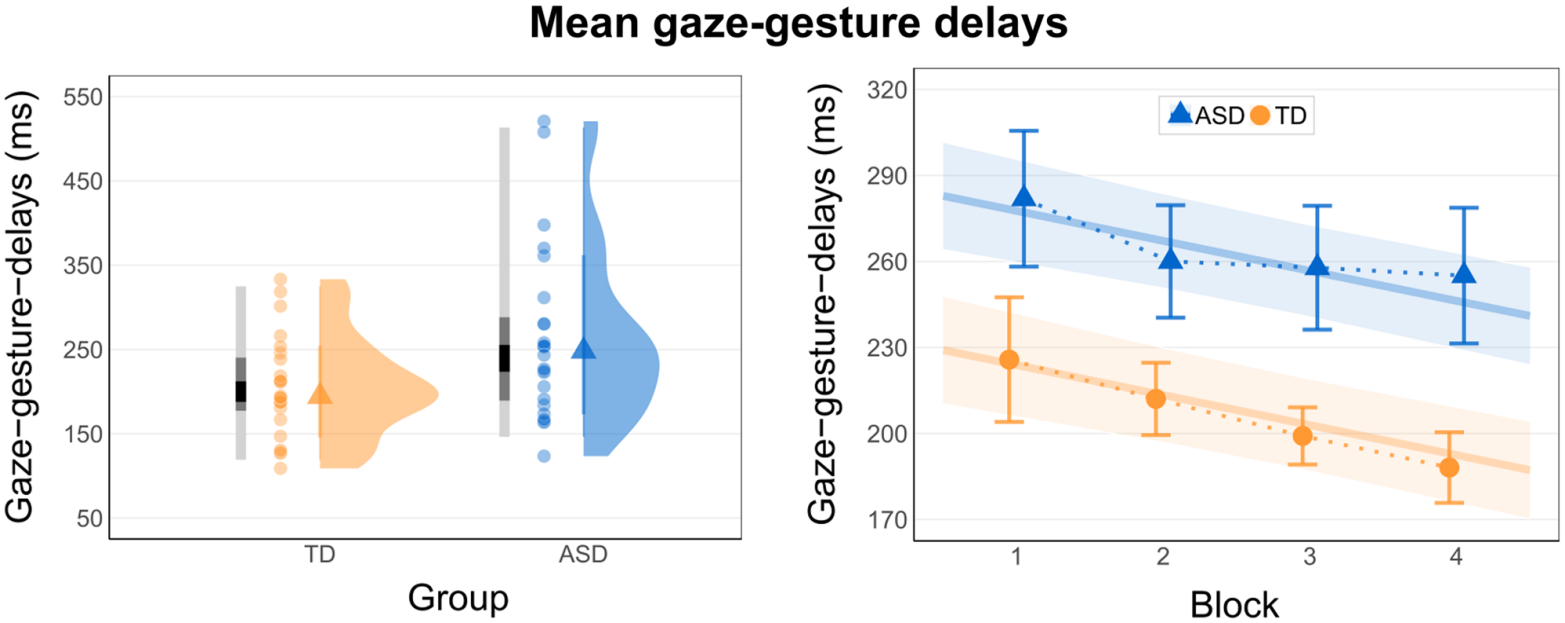
Gaze-gesture delays in both samples (left panel) and in both samples and experimental blocks (right panel). The delays represent the time in milliseconds from the onset of the communicative gaze shift to the onset of the pointing gesture*.* Left panel: Distribution of mean gaze-gesture delays aggregated for TD subjects in the comparison group in orange (left) and individuals with ASD in blue (right). Horizontally aligned density plots with medians (triangles), means per subject (point range), and the 25%, 50% and 95% colored confidence intervals (lightgrey = 95%; darkgrey = 50%; black = 25%). Right panel: Mean gaze-gesture delays of the ASD group in blue (triangles) and comparison group in orange (circles), separately for the four experimental blocks with standard errors of the means as error bars. Estimated linear regression lines for ASD group (blue) and comparison group (orange) with confidence bands displaying the standard error of estimates.

Gaze-gesture delays are depicted in Fig. 2 with descriptive statistics in Table 2. The delays represent the time in milliseconds from the onset of the communicative gaze shift to the onset of the pointing gesture. The likelihood ratio test revealed a significant increase in model fit by inclusion of group (*χ*^2^(1) = 5.67, *p* = .017), indicating larger gaze-gesture delays in the ASD group (*β* = 53.78 ms, 95% *CI* [11.08, 96.49], *p* = .014). Using the data from Table 2 in which repeated measurements were aggregated for subjects, the effect size of the group difference, indicated by Cohen’s *d* for independent, equally-sized groups was moderate to large (*d* = 0.69, 95% *CI* [− 1.272 to − 0.107]).

**Table 2:** Descriptive statistics of the IaPS measurements.

	M	SD	Md	min	max
**Gaze-gesture delays**					
ASD	264.05	103.13	248.05	123.45	520.70
TD	206.02	59.45	202.85	108.91	333.25
**SD of delays**					
ASD	95.19	44.64	81.15	42.05	251.6
TD	81.02	46.69	72.46	36.09	259.33

All values given in ms. Descriptive statistics for groups were retrieved from participant-wise aggregated data.

**Figure 3:**
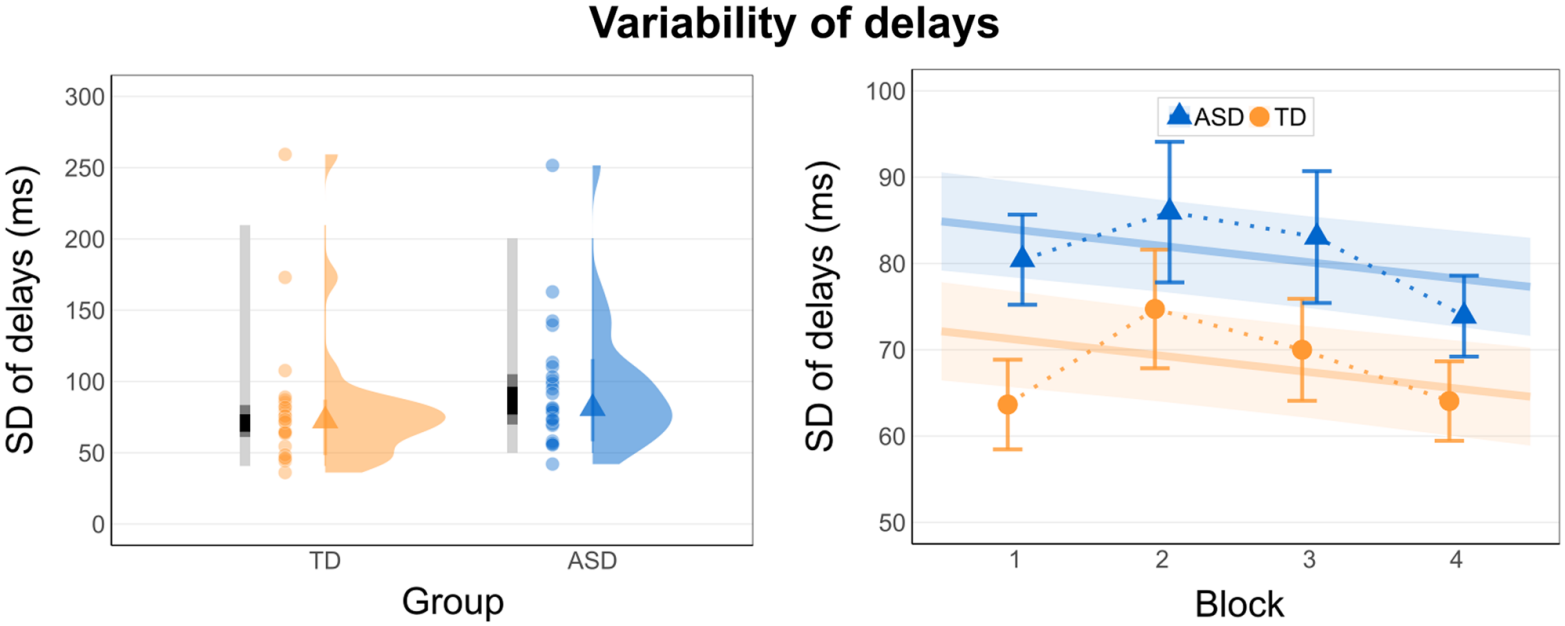
Within-subject SD of delays in both samples (left panel) and in both samples and experimental blocks (right panel). Left panel: Distribution of SD of delays aggregated for TD subjects in the comparison group in orange (left) and individuals with ASD in blue (right). Horizontally aligned density plots with medians (triangles), means per subject (point range), and the 25%, 50% and 95% colored confidence intervals (lightgrey = 95%; darkgrey = 50%; black = 25%). Right panel: SD of delays of the ASD group in blue (triangles) and comparison group in orange (circles), separately for the four experimental blocks with standard errors of the means as error bars. Estimated linear regression lines for ASD group (blue) and comparison group (orange) with confidence bands displaying the standard error of estimates (data in right panel is displayed after exclusion of extreme values in each group).

SD of delays are depicted in Fig. 3 with descriptive statistics in Table 2. Closer inspection of the data revealed seven extreme values, which were defined as values above the third quartile + 3 × inter-quartile-range or below first quartile – 3 × inter-quartile-range, calculated per group (We conducted a similar extreme value identification for the data of mean gaze-gesture delays and found 18 extreme values in the ASD group (0.8% of data) and 17 extreme values in the TD group (0.7% of data). Exclusion of these values did not change the pattern of results or improved model diagnostics.). Extreme cases were four observations of one participant from the ASD group and one and two observations from two TD participants (1.8% of data). Excluding these cases led to a reduction of the residual standard error and an adjustment of normality of residuals in the model. Comparison of trimmed models revealed a marginal increase in model fit by inclusion of the group factor (*χ*^2^(1) = 2.90, *p* = .091), and a small effect size for an increased SD of delays in the ASD group (*β* = 12.73 ms, 95% *CI* = [− 2.14, 27.60], *p* = .093).

### Correlation analysis

To determine whether the increased temporal delays between gaze and gesture onsets were associated with a greater within-subject variability in the temporal alignment, correlations of the IaPS indices were calculated. With regard to the relationship between the measurements , we found a significant positive correlation, indicating that individuals with larger gaze-gesture delays also produced larger SD of delays (*r* = .68, 95% *CI* = [.49, .81], *p* < .001). This was true for participants with ASD (*r* = .82, 95% *CI* = [.62, .92], *p* < .001) as well as for TD participants (*r* = .51, 95% *CI* = [.14, .76], *p* = .010).

Exploratory correlation analysis of IaPS parameters with clinical screening instruments (AQ, EQ, SQ, SPQ, ADC, RME, BDI) in the ASD group revealed significant correlations of gaze-gesture delays with SQ scores and RME scores, indicating lower scores on RME (“Reading the Mind in the Eyes” test measuring mentalizing with emotional demand) in participants who produced larger delays (*r* = − .47, 95% CI = [− .73, − .08], *p* = .022) and higher systemizing scores for individuals with shorter delays (*r* = -.44, 95% CI = [− .72, − .04], *p* = .033). Please note that due to the exploratory nature of this observation, the *p*-values are reported without correction for multiple testing and would be non-significant after Bonferroni correction (see Supplementary Material, Supplementary Table 3 for all correlations and [non-] adjusted *p*-values).

### Trajectory analysis

Regarding the analysis of the trajectory data, a likelihood ratio test showed no improvement of model fit above chance level by inclusion of group as fixed factor (*χ*^2^(1) = 0.99, *p* = .637) indicating that participants with and without ASD produced similar gesture amplitudes (*β* = 24.57 mm, 95% *CI* = [− 22.44, 71.59], *p* = .306). Likewise, there was no significant effect of group on the mean velocities of the gestures, as indicated by a comparison of LMMs (*χ*^2^(1) = 0.05, *p* = .913), indicating that there was no difference between groups in the velocities of gestures above chance level (*β* = 0.007 mm/ms, 95% *CI* = [− 0.06, 0.07], *p* = .818).

## Discussion

Here we report a crucial measure of IaPS in an experimentally controlled version of a real-life interaction, allowing for a precise quantification of IaPS in a millisecond resolution. The inclusion of a clinical group of adults with an ASD diagnosis provided evidence for a shift of nonverbal temporal baselines of ~ 55 ms with a 95% CI of [11.08, 96.49] between groups*.* Whereas there was no significant group effect in the SD of delays, the results of the correlation analysis showed that enlarged gaze-gesture delays were associated with an increased intrapersonal variability of delays. As such, the two indices of IaPS should be considered as dependent. Thus, we infer that IaPS in ASD may be best described as enlarged and more variable temporal alignment of nonverbal signals, indicating a weaker temporal coherence of nonverbal signals within individuals. Within the ‘weak central coherence’ theory of autism^81^, it is assumed that individuals with ASD show a local processing bias that entails differences in the integration of information compared to TD individuals. The interpretation herein represents a transmission of the weak central coherence account to the temporal domain^38^.

Our approach extends previous findings of asynchrony between gestures and semantic aspects of speech in ASD by de Marchena and Eigsti^6^ into the nonverbal domain and points to a transferability of increased binding windows in ASD^37^ into the social domain.

Furthermore, we found no evidence for systematic group differences in the amounts of communication behavior, which is in accordance with previous studies^1,3,7^. This lack of group differences further implies that the group differences in IaPS cannot be ascribed to differences in spontaneous communication habits or the spontaneous usage of different communication channels and their combinations.

We extracted spatio-temporal parameters from the gestures (i.e., gesture velocities and amplitudes) and conducted group comparisons. Our results do not support the assumption of altered spatio-temporal features of the gestures produced by individuals with ASD. Furthermore, as no group differences were found in the trajectory analysis, we can infer that the enlarged delays between gaze and gesture onsets were not driven by group differences in spatio-temporal features of the gestures.

Beyond that, the results of the exploratory correlation analysis indicate a relation of IaPS with mentalizing abilities and systemizing styles in ASD. However, these hypotheses need to be confirmed by future studies.

It could be assumed that the mechanisms of IaPS are acquired early in life in typical development. Before any verbal communication takes place, shared timing mechanisms in communication behavior are acquired through caregiver-child interactions^17–20^. Such temporal coordination of communicative behaviors during childhood is arguably a prerequisite for intuitive reciprocity in adulthood. It is possible that individuals with ASD are more likely to miss either acquisition or fine-tuning of IaPS mechanisms that persist into adulthood, as supported by our results. Such learning of temporal structures of communication obtained in infancy is also supported by early childhood intervention studies in autism. Despite reported variability between intervention studies^82,83^, meta-analyses show that training social exchanges with caregivers or therapists has positive effects^82–86^. For example, Sandbank et al.^86^ report positive outcomes from behavioral, developmental, and naturalistic developmental behavioral interventions (NDBI), all of which involve different forms of reciprocal interaction training to learn temporal structures of communication (e.g., turn-taking, imitation, joint-attention). Rodgers et al.^83^ also report positive effects of NDBI on adaptive behavior and cognitive skills in a follow-up survey 2 years after intervention. Bejarano-Martín et al.^84^ report a medium effect size of focused interventions on social and communication skills, with effects being stronger for younger children and more sessions.These studies could be considered as evidence that early support for interactions with children with ASD promotes learning of temporal coordination strategies between and within individuals.

Different baselines of IaPS in TD and ASD, as we presented it, could have an impact on interactions as it has been suggested that gaze needs to be coordinated with other communication signals within individuals for successful reciprocity^29^. Our results imply different nonverbal signal coordination mechanisms between adults with and without ASD, whereas shared mechanisms are potentially a basis for communicative success and interpersonal alignment^14,15,29^. In this sense, different baselines of IaPS display a potential key mechanism that may explain reduced interpersonal synchrony between individuals with and without ASD.

Regarding the concept of predictive coding in the context of social interaction, Koban, Ramamoorthy, and Konvalinka^87^ argue that interpersonal synchronization takes place due to the cognitive principle of optimization. Accordingly, people align their behavior in order to achieve a minimization of prediction errors. Regarding a more common baseline of IaPS in TD adults, prediction errors will probably less likely occur between those individuals with shared and more stable temporal integration of signals, which potentially facilitates nonverbal alignment. Contemplating an enhanced emphasis on prediction errors in ASD, as postulated by van de Cruys et al.^88^, it could be assumed that in social interactions of dyads with and without ASD, reciprocal violations of predictions that are outside the individual windows of expected uncertainty occur more frequently. Possibly, this is due to differences in perceptual processes; the perception of individuals with ASD could be modulated by inflexible processing of prediction errors^89^, whereas the perception of TD individuals could be shaped by signal encodings of the interaction partner that fall outside an expected range. Further research in interactive dyad settings chould be conducted in order to test these assumptions.

Prospectively, measures of IaPS could inspire improvements of diagnostic procedures for autism in adulthood. If future studies are able to replicate findings, generalize them to further nonverbal domains, and indicate them as specific to ASD, it may be possible to develop a diagnostic tool based on quantitative parameters of IaPS that aids objective assessments of ASD in adulthood. It should be noted, however, that in the exploratory correlation analysis we did not find a relationship between autism trait strength (AQ) and IaPS (see Supplementary Material, Supplementary Table 3). Because the AQ encompasses autism traits outside of social communication (e.g., imagination, attention to detail, attentional switching) and is not specific to ASD (see discussion in Koehler et al.^2^ and Wigham et al.^89^), future studies should include alternative scales that are specific to communication difficulties in autism to assess possible associations.

There are some limiting aspects of this study that need to be considered. We chose to quantify IaPS in a real-life interaction task but aimed to confirm a high standardization. It is unclear how our findings generalize to more naturalistic scenarios in which even more signals beyond gaze and pointing need to be intrapersonally coordinated and in which the signals of the interaction partner are more dynamic. We would assume that differences in IaPS between individuals with and without ASD also appear during scenarios with increased communicative complexity, but future studies need to clarify this assumption.

Furthermore, the finding of equal quantities of spontaneous communication channel usage must be considered in the light of the experimental setup. As the task was repetitive and structured and the participants were seated with their head fixated, the repertoire of communication was potentially reduced. As this reduction applied equally to both groups, our results are important for our implications, yet they need to be contrasted to studies that used open, non-structured interactions^1,3,8^.

We used multilevel modelling to test the group effect for significance. Multilevel modelling makes it possible to control for random variation by subjects that could in case of no consideration bias results and inhibit successful replication of results. The application of mixed models decreases Type-I error rates compared to ANOVA^78,79^. Beside these merits, it must be noted that the interpretation of significance of results based on *p*-values was criticized^90^ and should always be accompanied by a consideration of effect sizes and confidence levels.

Furthermore, the ASD sample in the current study included individuals who all received a F84.5 diagnosis. As such, it is unclear how the results translate into other domains of the autism spectrum.

We strive for unbiased language in the context of autism and used person-first language based on considerations in^91^.

## Conclusion

The temporal coordination of communication signals within individuals (i.e., IaPS) is assumed to be an integral feature of social interactions. We measured the temporal alignment of gaze and gesture signals within individuals during a real-life interaction task in adults with ASD and a comparison group of TD adults. The results of this study support the assumption of different temporal fine-tuning of nonverbal behavior between adults with and without ASD, which was expressed as weaker temporal coherence between nonverbal signals in individuals with ASD. This shift of temporal baselines potentially affects social interactions between individuals with and without ASD and represents an objective behavioral marker of communication in adults with ASD that may be useful for diagnosis and target of treatments. Future studies should investigate the consequences of differences in IaPS on communication quality, interpersonal alignment, and social impression formation in addition to investigations of the specificity of the effect to autism.

## Supplementary Information

Supplementary Information.

## Data Availability

Primary data of this study are not openly available as they contain information that could compromise patient privacy according to the applicable general data protection regulation (DSGVO, 2018). Specific referenced data are available from the corresponding author upon reasonable request (i.e., specific additional analyses, meta-analyses, and replication) from researchers wishing to use them for non-commercial purposes, without breaching participant confidentiality. The scripts that were used for data acquisition and analysis are available at the Open Science Framework (https://osf.io/t4wmr/).

## Abbreviations

ASD: Autism spectrum disorder
IaPS: Intrapersonal synchrony
TD: Typically-developed
IQ: Intelligence quotient
AQ: Autism quotient
EQ: Empathy quotient
SQ: Systemizing quotient
BDI: Becks depression inventory
SPQ: Sensory perception quotient
ADC: Adult dyspraxia checklist
WST: Wortschatztest
WIE-III: Wechsler intelligence scale adults III
RoI: Region of interest
LMM: Linear mixed model
GLMM: Generalized linear mixed model
RME: Reading mind in the eyes task
