# Supplementary material for: Intrapersonal synchrony analysis reveals a weaker temporal coherence between gaze and gestures in adults with autism spectrum disorder
Source: Sci Rep. 2022 Nov 27;12:20417. doi: 10.1038/s41598-022-24605-8 (PMC9701674; doi:10.1038/s41598-022-24605-8)
Supplement: Supplementary file 1 — Supplementary Information. [file 41598_2022_24605_MOESM1_ESM.pdf]

[Supplementary Material]

Intrapersonal Synchrony Analysis reveals a Weaker Temporal Coherence between Gaze and Gestures in Adults with Autism Spectrum Disorder

Authors: Carola Bloch, Ralf Tepest, Mathis Jording, Kai Vogeley, & Christine Falter-Wagner

S Figure 1. *Depression measures (x axis) against IaPS measures (y axis) in ASD group (N = 24)*

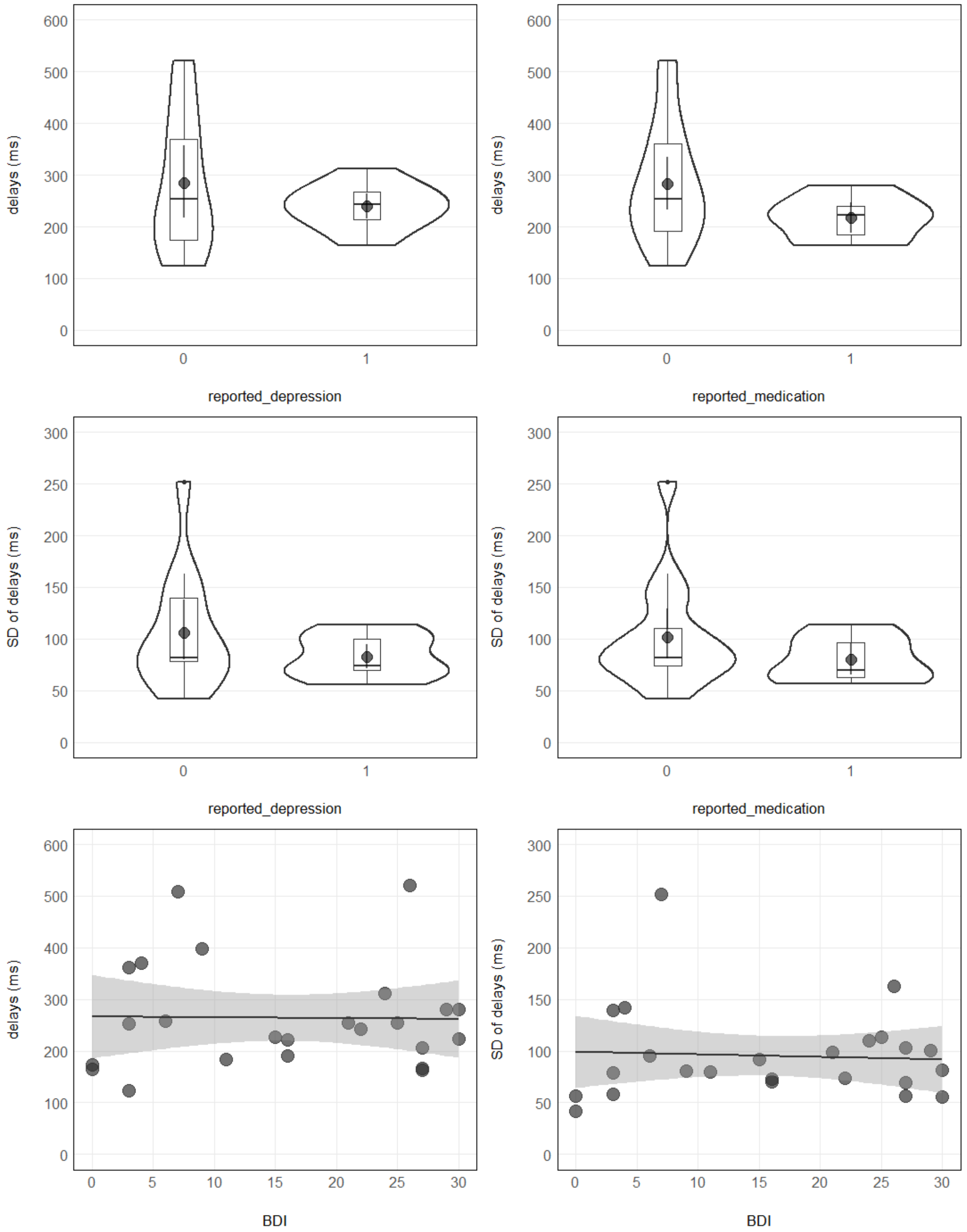

Note. **Upper panels:** Mean gaze-gesture-delays as violin plots with boxplots and mean values as points, separated for reported depression (0 = no reported depression; 1 = reported depression) (left) and reported medication with antidepressants (0 = no reported medication with antidepressants; 1 = reported medication with antidepressants) (right). **Middle panels:** SD of gaze-gesture-delays as violin plots with boxplots and mean values as points, separated for reported

depression (0 = no reported depression; 1 = reported depression) (left) and reported medication with antidepressants (0 = no reported medication with antidepressants; 1 = reported medication with antidepressants) (right). **Lower panels:** BDI scores against gaze-gesture-delays (left) and SD of delays (right) with fitted regression lines and confidence bands. Including BDI in the LMM predicting gaze-gesture-delays did not improve model fit ($\chi^2(1) = 0.27$, $p = .605$). Likewise, including BDI as a predictor in the LMM predicting SD of delays did not significantly improve model fit ($\chi^2(1) = 0.00$, $p = .994$).

S Figure 2. *IaPS measures (y axis) before and after Covid-19 setup modifications (x axis) in groups*

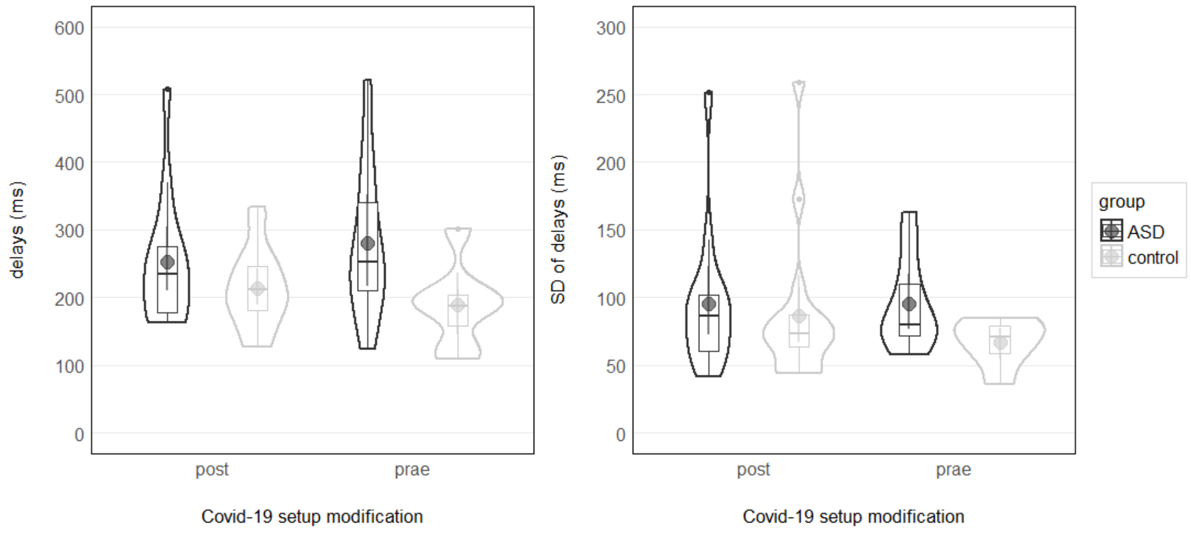

Note. **Left panel:** Gaze-gesture-delays as violin plots with boxplots and mean values as points, colored by groups and separated by Corona factor. **Right panel:** SD of delays as violin plots with boxplots and mean values as points, colored by groups and separated by Corona factor. Including the Corona factor in the LMM predicting mean gaze-gesture-delays did not improve model fit ($\chi^2(1)$ = 0.19, p = .662). Likewise, including the Corona factor as a predictor in the LMM predicting SD of delays did not significantly improve model fit ($\chi^2(1)$ = 0.15, p = .702).

Supplementary Table 1. *Number and percentage of gaze pathways in both groups in both task versions: Gaze pathway was either i) direct (from eye-contact fixation directly to target), ii) fix_random (additional fixation of a random region before fixation of target), or iii) fix_social (additional fixation of the social region before saccade to target)*

	direct		fix_random		fix_social	
	n	%	n	%	n	%
Free task version						
ASD	520	19.5	121	4.5	167	6.3
TD	1033	37.6	139	5.1	154	5.6
Nonverbal task version						
ASD	1749	62.8	346	12.4	140	5.0
TD	1857	66.7	268	9.6	203	7.3

Note. Percentage is given in relation to all possible trials after exclusions of first trials per block, and missing trials for free task version, per group.

S Figure 3. *IaPS measures (y axis) in three possible pathways of communicative gaze shifts (x axis) in both groups*

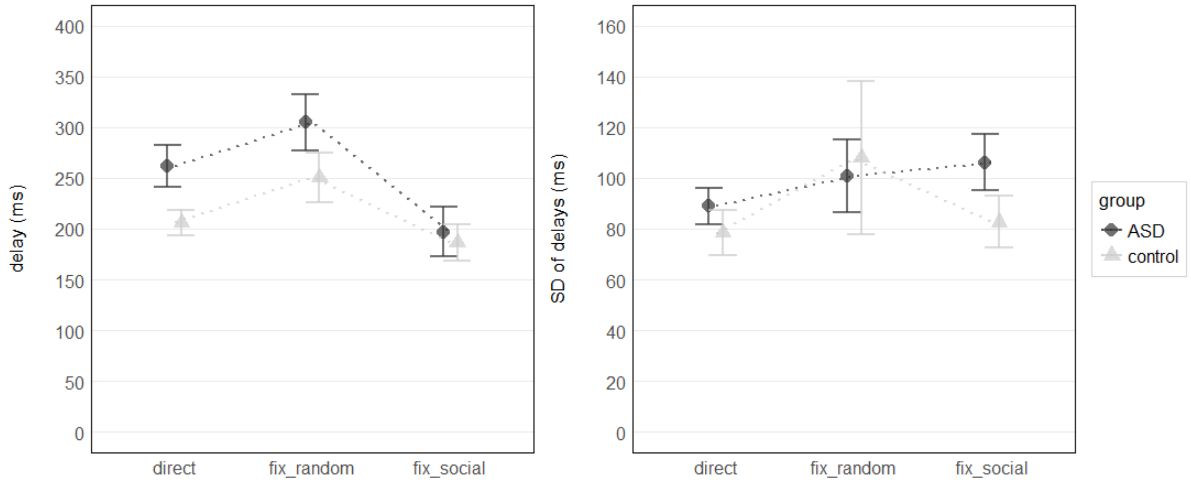

Note. **Left panel:** Mean gaze-gesture-delays in samples with standard errors for each sample in three factor levels of gaze pathway. **Right panel:** SD of gaze-gesture-delays for each sample in all factor levels of gaze pathway, values for fix_random missing for one participant in ASD group and three subjects in TD group, and values for fix_social missing for three subjects in ASD group and five subjects in TD group, all due to non-occurrence of > 1 trials, so SD could not be calculated.

Including pathway as a fixed factor in the LMM predicting gaze-gesture-delays significantly increased model fit ($\chi^2(2) = 135.01$, $p < .001$). Post-hoc pairwise comparisons revealed that all factor levels significantly differed. Pairwise comparisons (Holm-adjusted) revealed that gaze-gesture-delays with direct communicative gaze onsets were significantly smaller than those from gaze pathways with an additional fixation of the random region (estimate = 30.46, *SE* = 4.37, *z* = 6.97, *p* < .001) and significantly larger than those from gaze pathways with an additional fixation of the social region (estimate = -49.83, *SE* = 5.69, *z* = -8.76, *p* < .001). Delays with communicative gaze shifts with additional fixations of the social region were significantly smaller than delays with communicative gaze shifts with an additional fixation of the random region (estimate = -80.29, *SE* = 6.86, *z* = -11.71, *p* < .001). Including the interaction term group * pathway significantly improved model fit ($\chi^2(1) = 5.56$, $p = .038$), indicating that the impact of pathway on the delays differed between groups. Gaze shifts

with an additional fixation of the social region led in relation to the other pathways to a larger decrease in delays in the ASD group, see S4 panel A.

Considering the factor pathway as a possible influence on SD of delays, long data was aggregated for blocks and pathways per subject. A LMM that contained block and group as fixed factors and random intercepts for subjects was compared to a LMM containing pathway as additional fixed factor. The likelihood ratio test revealed no improvement in model fit above chance by inclusion of pathway as factor ($\chi^2(1) = 1.82$, $p = .403$). Neither did the inclusion of the interaction term group * pathway ($\chi^2(1) = 0.73$, $p = .695$).

Supplementary Table 2. *Number and percentage of excluded trials in different exclusion criteria (EC) for communicative gaze shifts per group in free task version (FT) and nonverbal task version (NT).*

	**EC1** n (%)	**EC2** n (%)	**EC3** n (%)	**EC4** n (%)	**EC5** n (%)	**EC6** n (%)	**EC7** n (%)	total n (%)
**FT**								
ASD	92 (3.3)	0 (0.0)	38 (1.4)	2 (0.1)	179 (6.5)	124 (4.5)	1519 (55.1)	1952 (70.7)
TD	96 (3.4)	5 (0.2)	14 (0.5)	35 (1.2)	48 (1.7)	170 (6.0)	1209 (42.5)	1554 (54.0)
**NT**								
ASD	96 (3.3)	5 (0.2)	59 (2.1)	63 (2.2)	117 (4.1)	58 (2.0)	247 (8.6)	645 (22.4)
TD	96 (3.3)	16 (0.6)	11 (0.4)	177 (6.2)	52 (1.8)	29 (1.0)	170 (5.9)	551 (19.1)

Note. Exclusion criteria (EC) from left to right: **EC1:** First trials of blocks; **EC2:** exclusions due to blinks < 100 ms before stimulus onset; **EC3:** saccade latencies < 75 ms; **EC4:** In FT rows: error trials due to technical issue, in NT rows: pointing preceded gaze; **EC5:** gaze was not in facial region of partner at stimulus onset; **EC6:** direct saccades to wrong target; **EC7:** more than one fixation before gaze landed on target or no fixation of target at all. Percentage is given in relation to all possible trials per group (for FT after exclusion of missing trials due to technical error).

Supplementary Table 3. *Exploratory correlation analysis of gaze-gesture delays with clinical screening instruments in the ASD group (n = 24).*

**Variables**	***r***	***p***	***p*.adj.**
AQ	-.24	.269	1
EQ	.04	.842	1
SQ	-.44	.033	.228
SPQ	.38	.070	.489
ADC	-.12	.585	1
RME	-.47	.022	.152
BDI	-.02	.941	1

Note. Pearson's correlation coefficients (*r*) with uncorrected *p*-values (*p*) and *p*-values after Bonferroni adjustment (*p*.adj.) with $\alpha = .05$. Correlations were calculated based on subject-wise aggregated data.

AQ = Autism Quotient; EQ = Empathy Quotient; SQ = Systemizing Quotient; RME = Reading the Mind in the Eyes test; SPQ = Sensory Perception Quotient; ADC = Adult Dyspraxia Checklist; BDI = Beck's Depression Inventory.

Supplementary Analysis 1. *Equivalence tests.*

Post-hoc equivalence tests were performed for non-significant group effects to provide an indication of the validity of the null hypothesis (H0). The function equivalence_test() of the parameters package was used to perform two one-sided tests (TOST) at the upper and lower bounds of a predefined region of practical equivalence (ROPE) (Lakens, 2017). The default ROPE was used for metric outcome variables ($\pm$ 0.1 of standardized parameters). For logistic models with binary outcome variables, it is suggested to manually adjust the ROPE range. Therefore, the ROPE for log odds coefficients was manually adjusted to a range of $\pm$ 2. Accordingly, H0 was accepted, if an effect and its 90% CI fell within the ROPE. H0 was rejected if the effect was statistically significant. If a non-significant effect and its 90% CI exceed the ROPE boundaries the question to whether accept or reject H0 was undecided.

Spontaneous channel use

Post-hoc equivalence tests for the models with summed uni-, bi-, and trimodal channel usage predicted by the group (ASD/TD) showed that with a predefined ROPE range of $\pm$ 2, the H0 (i.e., no group differences) for unimodal and bimodal channel use can be accepted (100% of the effects 90% CI fall into the ROPE). The equivalence test for trimodal channel usage yielded an undecided result, so H0 can neither be rejected or accepted (57.87% of the effects 90% CI fall into the ROPE).

SD of delays

An equivalence test for the trimmed model (i.e., extreme outliers excluded) showed that with a default ROPE of $\pm$ 0.1 of standardized parameters, the H0 for the group effect (i.e., no group differences) can be rejected (25.65% of the effects 90% CI fall into the ROPE).

Trajectory analysis

Post-hoc equivalence testing revealed that with a default ROPE of $\pm$ 0.1 of standardized parameters, it is undecided whether the null hypothesis for gesture amplitudes should be accepted or rejected (46.23% of the effects 90% CI fall into the ROPE). Likewise, an equivalence test revealed that with a default ROPE of $\pm$ 0.1 of standardized parameters, it is undecided whether the null

hypothesis for gesture velocities should be accepted or rejected (54.23% of the effects 90% CI fall into the ROPE).

Supplementary Literature

Lakens, D. (2017). Equivalence Tests: A Practical Primer for t Tests, Correlations, and Meta-Analyses. *Social Psychological and Personality Science*, *8*(4), 355–362.
